# Exploring Real-World Use of AI Chatbots for Mental Health Support: Cross-Sectional Survey Study

**DOI:** 10.2196/104316

**Published:** 2026-09-01

**Authors:** Sylvie Bernaerts, Fien Buelens, Nele A J De Witte, Andreas Balaskas, Toon Colman, Tom Van Daele

**Affiliations:** 1 Psychology and technology Centre of Expertise Care and Well-being Thomas More University of Applied Sciences Antwerpen Belgium; 2 School of Computer Science University College Dublin Dublin Ireland; 3 Centre for Technological Innovation Mental Health And Education Queen's University Belfast Belfast United Kingdom

**Keywords:** AI, chatbots, mental health, large language model, digital working alliance

## Abstract

**Background:**

In recent years, innovations in generative AI, in particular large language models (LLMs) in the form of AI chatbots, have found their way to the general public. First studies indicate a growing prevalence of individuals talking to AI chatbots about mental health–related topics; yet, knowledge of how, why, and which individuals are using these AI chatbots for their mental health is limited.

**Objective:**

This study aimed to provide insights into the use of AI chatbot–delivered mental health support.

**Methods:**

To do so, an online survey in 2 Belgian samples was conducted. A student sample was collected, and individuals who used AI chatbots for mental health support were included (approximately 40% of the student sample were eligible users). Second, a recruitment call for users of AI chatbots for mental health support was launched in the general public. Data from 349 participants, 276 members of the general public, and 73 students were included in the analyses.

**Results:**

Descriptive analyses were used to report the use of AI chatbot–delivered mental health support. Most respondents in the present samples were women and indicated having received or receiving professional mental health support. The most common conversation topics across both samples focused on personal and interpersonal issues. Freely accessible AI chatbots, mainly ChatGPT, were the predominant choice in both samples and were mainly chosen for their constant availability and accessibility. Respondents in the student sample also preferred their anonymity, and those in the general sample also used them because they felt supported by them. The preliminary associations found between digital working alliance and engagement factors in the student sample and in the general sample suggest that relational factors might play a role in sustained AI chatbot use but warrant further investigation.

**Conclusions:**

To conclude, this study confirms that general-purpose AI chatbots that are not designed or regulated for mental health support, specifically ChatGPT, are being used to obtain social and emotional support, often by individuals already familiar with professional mental health support.

## Introduction

Innovations in generative AI, in particular large language models (LLMs), have found their way to the general public in recent years. At their core, LLMs are enormous statistical language prediction machines that have been trained to recognize patterns in text and generate language that follows those patterns [1]. The latest generations of conversational agents or AI chatbots make use of such LLMs to generate dynamic, context-sensitive conversations that can feel very human-like to users. Ever since the release of generative AI chatbots, such as ChatGPT (OpenAI), Gemini (Google), Copilot (Microsoft), Claude (Anthropic), Meta AI (Meta) and Perplexity (Perplexity AI), people have been using these AI chatbots for work- and school-related tasks as part of their personal lives. Chatterji et al [2], for example, assessed approximately 1 million ChatGPT conversations from approximately 130,000 users between May 2024 and July 2025 and found that practical guidance (28.3%), writing (28.1%), and seeking information (21.3%) were the most common topics. The use of ChatGPT for relationships and personal reflection was less common, as it was addressed in 1.9% of conversations. More recently, Anthropic also analyzed 1 million Claude.ai conversations from March to April 2026 (approximately 639,000 conversations from unique users) and revealed that roughly 6% of conversations involved personal guidance, that is, seeking both information and perspective on what to do next [3]. Further exploring this personal guidance, Shen et al [3] categorized 10 clusters, of which health/wellness, relationships, and personal development accounted for 27.2%, 12.3%, and 6.3%, respectively, of personal guidance conversations. In other work assessing the affordances of AI chatbots in a large international sample of more than 5000 participants, Scherr et al [4] reported 5 specific functions of AI chatbots, namely, assistance for writing, coding, personal tasks, translation, and emotional assistance. Although people are using general-purpose AI chatbots mainly for specific tasks, a significant part of the population seems to be using them for mental health support as well [5-9]. These general-purpose AI chatbots are, however, different from mental health chatbots (with or without AI), which are conversational digital mental health interventions that use automated dialogue, either rule-based or AI-powered, to provide psychological support, psychoeducation, symptom monitoring, or evidence-based self-help strategies to users through text- or voice-based interactions.

The LLMs powering AI chatbots have been described as capable of cognitive empathy, recognizing emotions, and providing emotionally supportive responses in various contexts [10]. In addition, their anthropomorphic design [11] and sycophantic nature [12], which involve affirming users’ feelings and behaviors, may make them appealing for mental health support. Although data on prevalence are limited, first survey studies in a general US adult sample have examined the use of LLMs for mental health support. Stade et al [6] found that 24% of participants reported LLM use for mental health support, while Ueda et al [13] found that 35% of respondents reported using AI tools at least once a week for mental health support. In another study assessing individuals with an ongoing psychological condition, Rousmaniere et al [5] reported that nearly 49% of participants had used AI chatbots for psychological support. These findings warrant caution since these general-purpose AI chatbots were not created for this use and have also not been adequately validated for safety or effectiveness. Financial cost, stigma, and lack of access are well-known barriers to mental health support [14-16], and digital interventions such as mental health chatbots have long been proposed and evaluated as scalable and accessible solutions [17,18]. Initially, mental health chatbots such as Woebot (Woebot Health) or Wysa (Wysa) were mainly based on decision trees and rule-based systems [19]. Systematic reviews revealed mixed results, but these mental health chatbots have generally been found to reduce symptoms of depression and general distress [17,20]. More recent mental health chatbots and updated versions of existing ones claim to use AI, specifically LLMs, to generate more human-like dialogue [19]. For example, the first randomized controlled trial assessing the efficacy, user engagement, acceptability, and therapeutic alliance of a 4-week intervention with Therabot (Dartmouth College), a generative AI chatbot trained on cognitive behavioral techniques, showed promising results after the intervention and additional 4-week follow-up [21]. The study demonstrated reductions in symptoms of depression, anxiety, and eating disorder, as well as good user engagement and high user ratings [21]. Recent systematic reviews are, however, not conclusive on the efficacy of AI mental health chatbots [20,22-24]. While some reviews showed that AI-powered mental health chatbots are effective in reducing symptoms of depression and anxiety compared to control conditions [20,22,23], another review mainly confirmed their feasibility and acceptability, yet assessed effectiveness as inconclusive [24]. One interesting factor that might have influenced these mixed findings is therapeutic or working alliance, a mutual collaboration and partnership between therapist and client [25]. The working alliance has proven to be central in human-delivered mental health support [25] and the concept has also recently been applied to digital health products. For example, working alliance has been associated with engagement with and clinical outcomes of an unguided mobile mental health app [26]. Regarding AI chatbots, Heinz et al [21] showed, for the first time, that individuals were able to develop a working alliance with an AI chatbot (Therabot). There is, however, still much debate on the safety of using this technology for mental health purposes, in particular regarding topics such as crisis management and suicidality or privacy and confidentiality [27]. The advancements in mental health chatbot architecture due to rapid AI innovations have thus left the field with an evidence base characterized by variable clinical evidence and evaluation rigor [19].

Notwithstanding the evidence base highlighting the potential of specific mental health chatbots, the use of general-purpose AI chatbots for mental health support is empirically underexamined. Most studies to date have focused on answering the question of whether AI mental health chatbots based on LLMs can perform mental health tasks, whereas accumulating evidence indicates that people are rather steering toward general-purpose AI chatbots because of their availability and ease of access [6]. Research on the use of these chatbots for mental health purposes is limited, yet rapidly growing [3,5,6,8,28]. Studies that do focus on real-life use of LLM-powered chatbots for mental health have mostly been interview-based qualitative studies assessing how people engage with these chatbots and how they perceive such interactions. These studies often reported favorable results describing perceived effectiveness, usability, and emotional dynamics, but also challenges and limitations [29-33]. Nevertheless, knowledge about the scale of this behavior remains limited. Such knowledge, combined with insights into user characteristics and use patterns, is nevertheless essential to inform ethical, clinical, and policy decisions in this field. This study therefore aimed to assess how, why, and which individuals are using AI chatbots for mental health support, in particular general-purpose AI chatbots. In addition, we explored whether a digital working alliance, defined as the therapeutic relationship between a client and software [34] or, in the present study, between an individual and an AI chatbot, was associated with AI engagement characteristics.

## Methods

### Study Design

To gain insights into the use of conversational AI for mental health support, we developed an online survey assessing participants’ use of AI chatbots for mental health purposes. Two samples of participants who used AI chatbots for mental health support were recruited: from the general population and among students of the Applied Psychology program at Thomas More University of Applied Sciences, both in Belgium.

### Participants

Both samples were recruited through convenience sampling. The student sample was recruited through an internal course credit program for applied psychology students with invitations disseminated via the official program platform. In the student sample, 181 individuals gave informed consent to participate in the study. After applying the eligibility criterion of actual experience using AI chatbots for mental health support, 108 were excluded from the study due to lack of experience, leaving 73 participants (10 men and 63 women) in the student sample with a mean age of 19.75 (SD 2.52) years, ranging from 18 to 27 years. Five participants had an academic or professional bachelor’s degree, and 68 had a secondary school certificate. There were no missing values in the student sample.

This sample was complemented by the recruitment of members of the general public with experience in using any AI chatbot for mental health purposes. These were recruited through open calls disseminated via social media (ie, LinkedIn [Microsoft Inc]), written media (ie, at the end of newspaper and magazine articles), and during lectures and seminars. There were no exclusion criteria. In the general population sample, 277 individuals were recruited. Data from 276 participants were used in this study. One participant was excluded due to no actual experience using AI chatbots for mental health support. Data of 23 participants were incomplete due to drop-out, resulting in missing data for the following variables: helpfulness, negative experiences, and advising the use of AI chatbots (all n=4), Digital Working Alliance Inventory (D-WAI; n=10), being in treatment or having received treatment (n=13), the sociodemographic data, such as age, gender, and degree (n=23). Missing values were handled using pairwise deletion to maximize data usage. The general population sample (195 women, 54 men, 1 nonbinary participant, 2 participants who did not report their gender, and 23 with missing data) had a mean age of 45.46 (SD 14.58) years, ranging from 19 to 82 years. Ninety-two participants had an academic or professional bachelor’s degree, 74 had a master’s degree, 74 had a secondary school certificate, 7 had a doctoral degree, and 6 had a primary school certificate (missing, n=23).

### Data Collection

The online survey was created using QuestionPro (QuestionPro Inc) and was completed by participants between October 2025 and March 2026. The survey consisted of 26 questions, comprising both closed- and open-ended questions. The survey is available in the original Dutch version and a translated English version (see Multimedia Appendix 1). Participants answered multiple-choice questions accompanied by free-text options. The survey involved questions concerning how, why, and which AI chatbots participants used for mental health purposes: (1) the extent to which conversations with AI chatbots were considered helpful, (2) whether they would advise the use of AI chatbots, (3) whether they had negative experiences with AI chatbots, (4) technical aspects such as modality, medium, and duration, and (5) personal characteristics, such as whether participants were receiving psychological treatment. Participants were allowed to give multiple answers to the questions concerning how and why they had used AI chatbots. The questions were set up and discussed by the authors based on questions from clinical practice and existing research and were intended to allow for descriptive analyses. To the best of our knowledge, no validated questionnaires were available to gain insight into the topics surveyed at the time of designing the study protocol. These questions were complemented by a translated version of the D-WAI [34]. This study aimed to explore the working alliance between the participant and the AI chatbot and its relationship with AI chatbot engagement, using the D-WAI. The D-WAI is a 6-item questionnaire, with each item rated from 1 (strongly disagree) to 7 (strongly agree) and summed to generate a total score from 6 to 42. The D-WAI was translated from English to Dutch using a forward-backward translation process, performed by 4 authors (TVD, NAJDW, FB, and SB) who held C1-level English certification. Authors TVD and NAJDW independently translated the D-WAI from English to Dutch and discussed the items to reach consensus on the initial Dutch version. Then authors FB and SB independently translated the Dutch items back to English. FB and SB were not aware of the original English items. The 4 involved authors then discussed discrepancies and adjusted the Dutch items until consensus was reached regarding the translation and the preservation of the intended meaning of the items. The translated version was not pilot tested, and no psychometric properties were assessed before use in the present study. The original D-WAI has been shown to have satisfactory psychometric properties [35] and the reliability of the translated instrument in this sample was acceptable in the student sample (Cronbach α=0.73) and good in the general population sample (Cronbach α=0.83).

### Data Analysis

Data were analyzed using SPSS (version 31; IBM Corp). Descriptive statistics (frequencies and percentages) were used to summarize survey responses and participant characteristics. Spearman Rho was used to assess the association between working alliance (D-WAI) and engagement (the frequency and duration of AI chatbot use). The 4 categories of the frequency or duration variables were coded from 1 to 4, with 1 representing the lowest frequency or duration and 4 representing the highest. The significance level was set at *P*<.05. Figures were created using R (version 4.2.2; R Core Team) and the following packages: *dplyr* [36], *stringr* [37], *ggplot2* [38], *cowplot* [39], *readxl* [40], and *tidyr* [41]. Qualitative data from the answers to the open-ended questions were categorized into topics in Dutch by authors FB and SB to facilitate summarization. FB and SB adopted an inductive coding approach and first categorized the data independently and discussed differences in categorization afterward until consensus was reached. Multiple codes were permitted and no predefined coding scheme was used. No intercoder agreement was calculated. Final codes were translated into English by the author SB.

### Ethical Considerations

The study was approved by the local ethics committee of the Applied Psychology program at Thomas More University of Applied Sciences (reference ECTP 2526_02). Participants were informed about the study’s topic, objectives, data management, and confidentiality procedures before starting the survey. All participants provided electronic or written informed consent prior to their involvement in the study. Data were collected anonymously; however, responses to the open-ended questions could have contained identifiable information. Therefore, the data were pseudonymized, and only pseudonymized data were archived. Participants in the student sample received a course credit corresponding to a score of 1/20 added to their exam score for a specific course. Participants from the general public did not receive compensation.

## Results

### Student Sample

Recruitment figures revealed that 40.4% (n=73) of the recruited first-year applied psychology student sample had some experience using AI chatbots for mental health purposes. More than half of students (n=42, 57.5%) reported either being in psychological treatment (n=21, 28.8%) or having received psychological treatment in the past (n=21, 28.8%). Of these participants, 83.3% (n=35) revealed having not informed their therapist or counselor about their conversations with AI chatbots.

**Figure 1:**
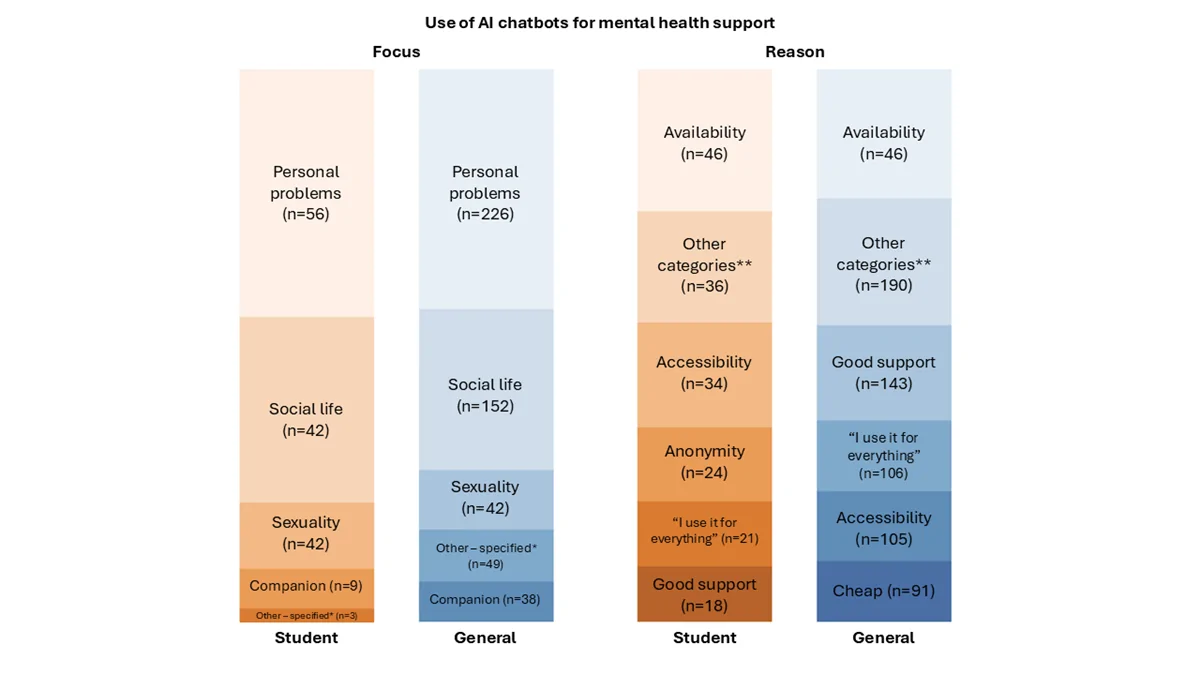
Participant usage characteristics.

**Figure 2:**
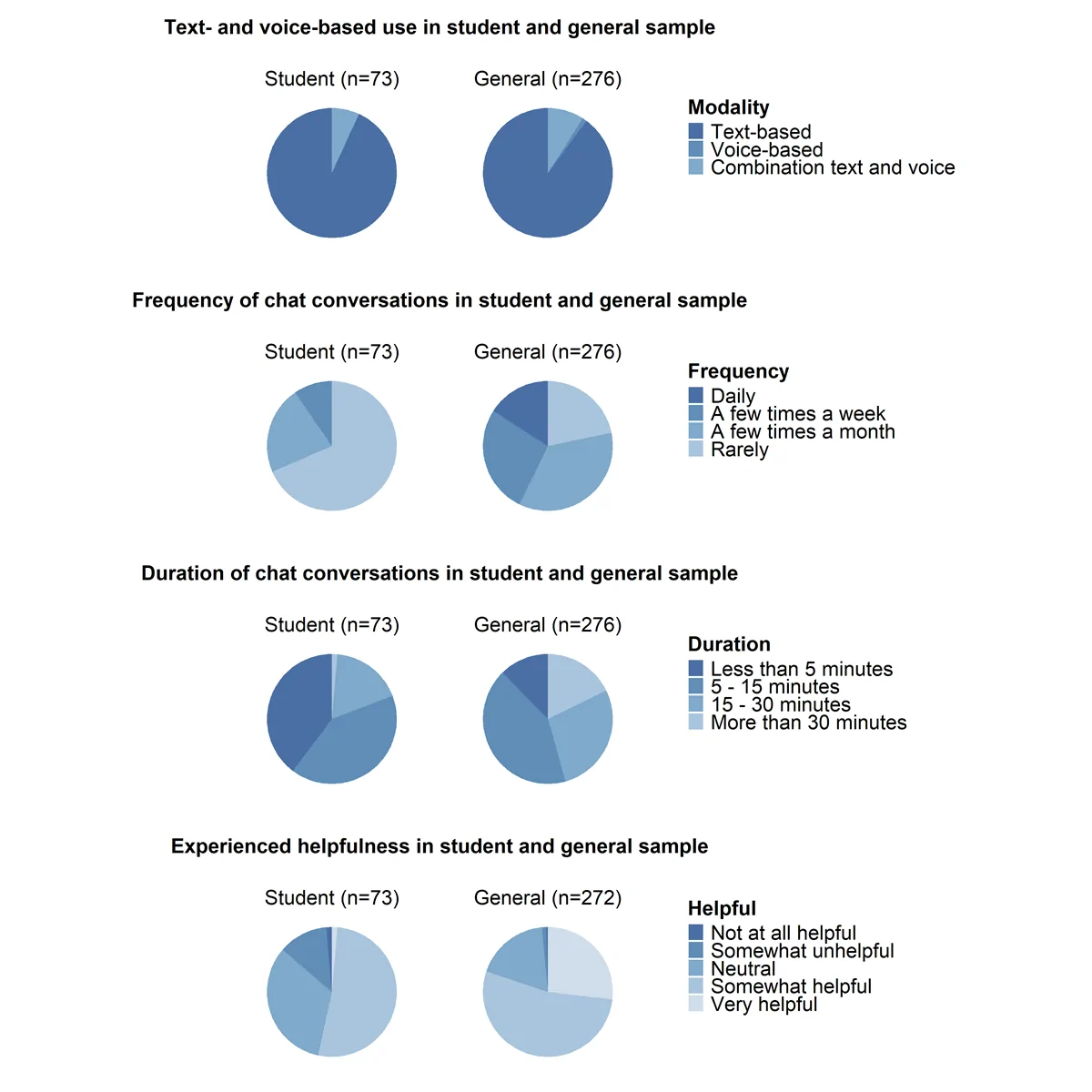
Modality, frequency, duration, and experienced helpfulness of AI chatbot use.

Student usage characteristics are presented in Figures 1 and 2, and Tables 1, 2, and 3. In this sample, most students used AI chatbots to talk about personal concerns or issues (n=56, 76.8%) and to talk about situations in their social lives (n=42, 57.6%). Approximately one-fifth of respondents used AI chatbots to discuss intimacy or sexuality (n=15, 20.6%), and a smaller part also indicated that AI chatbots were used for companionship or to feel less lonely (n=9, 12.4%). A minority of participants shared additional functions (n=3, 4.2%; see Table 1), which mainly involved relying on AI chatbots as empathic sounding boards and for emotional self-regulation.

**Table 1:** Additional focus topics for AI chatbot use.

Focus topics	Student (n=3)^a^	General (n=49)^a^
Empathic sounding board	3 (4.1)	15 (5.4)
Health or medical information tool	—^b^	15 (5.4)
Emotional self-regulation and self-development	—	6 (2.2)
Suicide prevention	—	1 (0.4)

^a^Numbers represent reported responses. Participants were allowed to give multiple answers.

^b^Not available.

The most popular AI chatbots to converse with were ChatGPT (n=70, 95.9%) and Snapchat’s My AI (n=19, 26%), and less commonly used AI chatbots were Copilot (n=9, 12.3%), Gemini (n=4, 5.5%) and DeepSeek (n=3, 4.1%). Other AI chatbots can be found in Table 2. Most participants used freely accessible AI chatbots (n=66, 90.4%), and a minority had a paid subscription (n=4, 5.5%) or had access to AI chatbots through school or work (n=3, 4.1%). Smartphones (n=64, 87.7%) and personal computers (n=36, 49.3%) were most often used as the conversation medium, whereas the tablets were the least commonly used option (n=4, 5.5%). Most students had only text-based interactions (n=68, 93.2%), although some used a combination of text and voice (n=5, 6.8%). None of the participating students reported daily mental health interactions with AI chatbots. A small proportion (n=7, 9.6%) of students had weekly mental health-focused interactions, 21.9% (n=16) had monthly interactions, and most students rarely used AI chatbots for mental health conversations (n=50, 68.5%). The most common conversation duration was 5-15 minutes (n=30, 41.4%), followed by less than 5 minutes (n=29, 39.7%), 15-30 minutes (n=13, 17.8%), and more than 30 minutes (n=1, 1.4%). The majority of students had been talking to AI chatbots for mental health purposes for at least 6 months (for 6 months: n=27, 37%; for a year: n=19, 26%; for more than 1 year: n=10, 13.7%), while others had started using them more recently (since 3 months: n=9, 12.3%; less than a month: n=8, 11%).

**Table 2:** AI chatbots used for mental health support.

Chatbot	Students (n=73)	General public (n=276)
Character.AI	1 (1.4)	2 (0.7)
ChatGPT (OpenAI)	70 (95.9)	239 (86.6)
Claude (Anthropic)	— ^a^	8 (2.9)
Copilot (Microsoft)	9 (12.3)	41 (14.8)
DeepSeek	3 (4.1)	3 (1.1)
Gemini (Google)	4 (5.5)	52 (18.8)
Grok (SpaceXAI)	1 (1.4)	3 (1.1)
My AI (Snapchat)	19 (26)	4 (1.4)
Perplexity	—	4 (1.4)
Replika	—	7 (2.5)
Other^b^	—	11 (4)

^a^Not available.

^b^Other chatbots: Abby, Ecosia AI, Meta AI, Spicychat.ai, Pi, Liven, Lumo, Le Chat (Mistral), Nomi AI, The Architect, unknown project-specific AI chatbot.

With respect to the reasons why AI chatbots were used, most respondents indicated their constant availability (n=46, 63%), followed by accessibility (finding it easier to talk to an AI chatbot than to a real person, n=34, 46.6%), anonymity (n=24, 32.9%), and using AI chatbots for everything, so also mental health (n=21, 28.8%). About a quarter of the students indicated that AI chatbots offered good support (n=18, 24.7%), that they were free or cheaper than professional services (n=14, 19.2%). A minority responded that they simply like talking to AI chatbots (n=9, 12.3%) or that they had been advised to use AI chatbots (n=3, 4.1%). Some participants also provided additional reasons (n=10, 13.7%), such as using AI chatbots as an add-on to professional help, their compliance, and a perceived lack of alternatives, among repetitions or elaborations of previously chosen categories (see Table 3).

Most students had a positive attitude toward mental health-focused conversations with AI chatbots, as more than half of the participants considered them to be rather helpful (n=38, 52%) or very helpful (n=1, 1.4%), while 12.3% (n=9) of students found them rather unhelpful and only 1.4% (n=1) very unhelpful. One-third of student participants (n=24, 32.9%) had a neutral attitude. Negative experiences were uncommon, with only a few students having experienced negative situations (n=3, 4.1%), mainly involving feeling misunderstood and experiencing sycophancy (extreme and insincere flattery). Nonetheless, the majority of students were unsure about recommending AI chatbot use for mental health purposes (n=31, 42.5%) or would probably not recommend it (n=23, 31.5%), whereas only 16.4% (n=12) would probably recommend its use (definitely would not recommend: n=6, 8.2%; definitely would recommend: n=1, 1.4%).

With respect to the digital working alliance, the mean total D-WAI score was 26.37 (SD 4.91). D-WAI was positively associated with frequency (small to moderate effect, Spearman ρ=0.36; 95% bootstrap CI 0.15-0.54; *P*=.002; n=73) and duration (small to moderate effect, Spearman ρ=0.33, 95% bootstrap CI 0.12-0.51, *P*=.004, n=73), meaning that higher working alliance scores were associated with higher frequency and longer duration of AI chatbot use (no causal direction was inferred). However, these findings should be interpreted with caution due to the heavy imbalance in observations and one empty category and limited variation in the categorical variables (see data mentioned above).

**Table 3:** Additional reasons for AI chatbot use.

Reason			Student (n=36)^a^		General (n=190)
Answer options			n=26		n=123
	Free or cheaper than official care	14 (19.2)		91 (64.7)	
	Anonymity	24 (66.7)		71 (25.7)	
	Recommended by others	3 (4.1)		8 (2.9)	
	I like talking to chatbots	9 (12.3)		44 (15.9)	
Open answers			n=10		n=67
	Quick availability	3 (4.1)		15 (5.4)	
	Compliance and docility	2 (2.7)		12 (4.3)	
	Not bothering anyone	1 (1.4)		5 (1.8)	
	Add-on to professional help	1 (1.4)		4 (1.4)	
	Better than professional support	—^b^		4 (1.4)	
	Experience of no alternative to AI chatbot	1 (1.4)		4 (1.4)	
	Control mechanism	1 (1.4)		1 (0.4)	
	To gain new insights	—		2 (0.7)	
	Irrelevant^c^	1 (1.4)		20 (7.2)	

^a^Numbers represent reported responses. Participants were allowed to give multiple answers.

^b^Not available.

^c^Participant input (Student n=1 and General n=20) to the question was not an answer to the question “Why are you using AI chatbots for mental health support?” or was not related to mental health support.

### General Population Sample

Because only individuals from the general public who had used AI chatbots for mental health support were recruited, we could not estimate the prevalence of AI chatbot use for this purpose in the general population. More than 3-quarters of participants reported either being in psychological treatment (n=111, 42.2%) or having received psychological treatment prior to this study (n=105, 39.9%), of which 64% (n=71) and 88.4% (n=92, missing n=1) indicated having not informed their therapist or counselor about their conversations with AI chatbots.

Usage characteristics of the general population sample are presented in Figures 1 and 2, and Tables 1, 2, and 3. Most participants used AI chatbots to talk about personal concerns or issues (n=226, 81.9%) and to talk about situations in their social lives (n=152, 55.1%). Approximately, one fifth of respondents also used AI chatbots to discuss intimacy or sexuality (n=56, 20.3%) and a smaller part also indicated that AI chatbots were used for companionship or to feel less lonely (n=38, 13.8%). Some participants also provided additional or more specific functions (n=49, 17.8%; Table 2), which mainly involved the use of AI chatbots as an empathic sounding board for brainstorming, venting, or exchanging ideas: “To talk about sadness and injustice in my life” and “Advice and reassurance concerning certain thoughts.” Some also reported using AI chatbots as a health or medical information tool or to help with explaining mental health information: “Questions about medication, advice for good self-care,” and “Diagnoses, symptoms, and therapies.” Others also pointed out that they use it as a support tool for emotional self-regulation and self-development: “To challenge myself and my thoughts, and to connect. For example, I feel bad about X. Is that correct? What can I do to get out of it?”

The most popular AI chatbots were ChatGPT (n=239, 86.6%), followed by Gemini (n=52, 18.8%) and Copilot (n=41, 14.9%). Other AI chatbots can be found in Table 2. Most participants used freely accessible AI chatbots (n=221, 80.1%) and a minority had a paid subscription (n=36, 13%) or had access to AI chatbots through school or work (n=19, 6.9%). Smartphones (n=221, 80.1%) and personal computers (n=109, 39.5%) were most commonly used as conversation media, and most respondents only had text-based interactions (n=248, 89.9%), while some used a combination of text and voice (n=25, 9.1%) or voice only (n=3, 1.1%). A total of 15.9% (n=44) of participants had daily mental health–related conversations with an AI chatbot, whereas 21.7% (n=60) rarely did so. The majority had either monthly (n=98, 35.5%) or weekly (n=74, 26.8%) AI chatbot interactions concerning mental health. Most participants estimated that their conversations on average lasted between 5 and 15 minutes (n=116, 42%). Other participants indicated a duration of less than 5 minutes (n=34, 12.3%), 15-30 minutes (n=77, 27.9%), or more than 30 minutes (n=49, 17.8%). More than half of the respondents had been talking to AI chatbots for mental health purposes for at least 6 months (for 6 months: n=100, 36.2%; for a year: n=46, 16.7%; for more than a year: n=29, 10.5%), while others have started using them more recently (since 3 months: n=70, 25.4%; less than a month: n=31, 11.2%).

With respect to the reasons for which AI chatbots were used, multiple responses could be selected. Most respondents were attracted by their constant availability (n=194, 70.3%), followed by feeling well-supported (n=143, 51.8%), accessibility (finding it easier to talk to an AI chatbot than a real person: n=105, 38%), and using AI chatbots for everything, including mental health (n=106, 38.4%). One third of participants also indicated the fact that they are free or cheaper than professional services (n=91, 33%) was a reason for use, as well as their anonymity (n=71, 25.7%). A total of 15.9% (n=44) responded that they just like talking to AI chatbots, and only 2.9% (n=8) were advised to use AI chatbots. A quarter of participants also provided additional reasons or specifications for their indicated reasons (n=67, 24.3%) in the open-ended response option (Table 3). Some participants emphasized AI chatbots’ quick availability: “They have quick access to information on the internet and can make connections lightning fast” or “It immediately pulls me out of deep slump moments.” Others used it for their compliance and docility, indicating “It is liberating and safe because they never judge” or “They are kind.” Some also use it as an add-on to professional help, “AI offers me support between therapy sessions. It helps me gain a clearer view of my inner world and make room for it.” Some even believe it to be better than professional support, “Better care, they analyze and explain what they notice. I get much more out of it than all the years of therapy combined.” Others use AI chatbots because they do not want to bother anyone with their issues, “I have no one in my immediate circle to talk to about it, or I don't want to burden those people with my worries.”

A total of 80.1% of participants (n=218) had a positive attitude toward mental health-focused conversations with AI chatbots, as more than half considered them to be rather helpful (n=145, 52.5%) or very helpful (n=73, 26.4%), while only 1.1% (n=3) and 0.4% (n=1) of participants found them rather unhelpful and very unhelpful, respectively (n=50, 18.4% had a neutral attitude; n=4, 1.5% was missing). Negative experiences were rather uncommon, with 11% (n=30) of respondents having experienced negative situations, mainly involving feeling misunderstood, sycophancy, experienced negative effects in real life, persisting unwanted topics or interactions, or providing incorrect information. A lot of participants indicated that they would probably (n=87, 32%) or surely (n=35, 12.9%) recommend the use of AI chatbots for mental health purposes, while the majority were unsure about it (n=99, 36.4%), 16.9% (n=44) would probably not recommend their use, and 2.6% (n=7) would definitely not recommend their use. data were missing for 4 participants (1.5%).

With respect to the digital working alliance, the mean total D-WAI score was 29.11 (SD 5.72). The D-WAI score was positively associated with frequency of use (small to moderate effect, Spearman ρ=0.26; 95% bootstrap CI 0.13-0.38; *P*<.001; N=266), meaning that a higher working alliance was associated with a higher frequency (no causation or direction inferred). There was no significant association between D-WAI and duration of AI chatbot use (Spearman ρ=0.03; *P*=.48; N=266).

## Discussion

### Principal Findings

This study assessed the usage characteristics of mental health support delivered by AI chatbots, in particular general-purpose AI chatbots, in a student sample and in the general public, in order to better understand why, how, and which individuals are using these chatbots. Forty percent of the initially recruited student sample reported having used AI chatbots for mental health support and were further inquired about their usage of this technology. This information was not available in the general population sample, as only individuals who used AI chatbots for mental health support were recruited. Most respondents in the present samples were women and reported having received or receiving professional mental health support at the time of the study. The most common conversation topics across both samples focused on personal and interpersonal issues. Freely accessible AI chatbots, mainly ChatGPT, were the predominant choice in both samples and were mainly chosen for their constant availability and accessibility. Respondents in the student sample also preferred their anonymity, and those of the general sample also used them because they felt supported by them. Both groups also indicated that they already used AI chatbots for other tasks, so mental health support was a logical addition. Participants mainly converse with general-purpose AI chatbots through text-based interactions on their smartphones. In general, the respondents found their interactions with these general-purpose AI chatbots helpful, and negative experiences were uncommon. To the best of our knowledge, our study also revealed, for the first time, an association between digital working alliance and general-purpose AI chatbot engagement, namely that in both samples a higher working alliance was associated with a higher frequency. In the student sample, a higher working alliance was also associated with a longer duration of AI chatbot use for mental health support, but not in the general sample (although no causal relation or direction is inferred). In the following paragraphs, we describe the findings in more detail and compare them to the current knowledge base.

### Respondents Used AI Chatbots for Mental Health Support

Recruitment numbers in the student sample revealed that 40% of our sample of first-year applied psychology students had some experience using AI chatbots for mental health support. This number contrasts the findings by Rackoff et al [28] in early 2024, indicating that only 5% of 428 US students (unknown educational track) had sought mental health support from an AI chatbot. Similar to the occurrence in the student sample, the prevalence of LLM use for mental health support in the general adult population seems to range from 24% to 47%. Li et al [42] found that 47% of 393 adult participants had used mental health AI chatbots. More recently, in 2 larger US adult samples of approximately 1800 respondents, Stade et al [6] and Ueda et al [13] revealed that 24% and 35% of participants had used LLMs for mental health purposes, respectively. In another US adult sample of 499 individuals with a past or present mental health condition, Rousmaniere et al [5] found that 49% of participants had used LLMs for mental health support. In a Dutch study exploring AI chatbot use in 771 adults with a (past) mental health condition, 38% of participants reported having used generative AI to talk about psychological complaints [43]. A recent estimation and narrative review revealed that 3%-70% of AI users use it for mental health purposes, with the authors approximating that 27% use it for mental health support [44]. More research in larger and more heterogeneous samples as well as a clear definition of mental health support is, however, needed to gain better insights into the prevalence of real-life AI chatbot use for mental health support [44].

In general, the survey responses revealed a predominantly female demographic. Most individuals engaging with the AI chatbots, more than half in the student sample and approximately 3-quarters in the general sample, had received or were receiving psychotherapy at the time of the study. Similarly, Zaia et al [33] also highlighted a predominantly female demographic of participants engaging with Character.ai’s Psychologist.ai chatbot. This study was, however, a qualitative study comprising a small sample size of 13 participants. The Dutch study performed by MIND had also revealed that nearly 78% of participants were women. The female demographic of both those and our participants might, however, reflect a recruitment bias rather than a true difference in users, considering participation in mental health–related studies might appeal more to women than to men. In contrast to our findings, Stade et al [6] found that users of LLMs for mental health were more likely to be younger, black and male. This study also revealed that users were more likely to report mental health diagnoses such as major depression or anxiety disorders. Although we did not assess or survey specific diagnoses, this latter result seems in line with our finding that a lot of participants were already either in mental health treatment or had received treatment.

With most student respondents rarely using AI chatbots for mental health conversations and one fifth having monthly interactions, AI chatbot use for mental health support appeared to occur relatively infrequently in this group. This notion seems to be supported by our findings that most conversations were rather short, lasting 15 minutes at most. Conversely, in the general sample, AI-powered mental health support seems more established, with the majority of participants having weekly or monthly interactions and a larger proportion of participants having longer conversations for up to 30 minutes. To date, multiple studies have found different frequencies of AI chatbot use for mental health. Zaia et al [33] found that most users engaged with Character.AI’s psychologist chatbot on a weekly basis for sessions varying in duration from less than 15 minutes to more than an hour. In addition, other studies also showed that daily and weekly conversation with AI chatbots for mental health is quite common among users [13,30]. Stade et al [6] found a monthly frequency of AI chatbot interactions for mental health. Interestingly, in both samples, most participants only started using AI chatbots for mental health support 6 months to one year prior to this study, while, for example, ChatGPT has been publicly available since November 2022.

In agreement with prior work [6,43], the vast majority of both samples used free AI chatbots, primarily ChatGPT. In the student sample Snapchat’s My AI was also quite common, and in the general sample Gemini was the second-most-mentioned AI chatbot. Since the majority of Snapchat users are teens and adolescents [45], it is not surprising that their AI chatbot was often reported in the student sample but not in the general population sample. In another study using interviews with a smaller sample, Siddals et al [30] found that Pi (Inflection) was most used, followed by ChatGPT. While most research has focused on assessing the safety and efficacy of (now AI-powered) mental health chatbots such as Woebot [46], Tess [47], or Wysa [48], these chatbots were not named by the participants in our study, nor in the Dutch MIND study [43]. One reason could be the language barrier, as these chatbots are not available in Dutch.

### AI Chatbots Use for Mental Health Support

With respect to the function of AI chatbot interactions for mental health, similar trends were revealed across both study samples. The multiple-choice answers showed that AI chatbots were primarily used to talk about personal concerns or issues, followed by social situations, intimacy and sexuality, and for companionship or to alleviate loneliness. Free-text responses from both groups revealed the use of AI chatbots as an empathic sounding board for brainstorming, venting, or exchanging ideas in a mental health context. These findings are in line with prior research [6,8,33]. Stade et al [6] found that most participants used LLMs because of the need for social and emotional support, but also to learn therapy skills and tools and to supplement existing therapy. Our findings add that this emotional and social support can cover both personal issues as well as interpersonal situations, intimacy and sexuality, or battling loneliness. Another study, exploring the perceived benefits, limitations, and use patterns of using Character.AI’s psychologist chatbot revealed that participants disclosed interpersonal challenges, anxious thoughts, and emotional distress [33]. Deeper exploration of the function of chatbot use revealed that the chatbot interactions were mainly used for emotion regulation, to ease acute emotional discomfort, and for deeper reflection, allowing more profound introspection, exploring behaviors, inner experiences, and relationships [33]. Consistent with Stade et al [6], our participants also indicated using AI chatbots for skills and techniques (eg, emotional self-regulation) and assessment and diagnosis (eg, understanding medical information).

Alongside all the aforementioned use, which can be considered “everyday mental health use,” there were, however, also examples of AI chatbots being used to either search mental health information or to aid interpretation of mental health information, which could be classified as use as a medical information tool. This is in line with findings from Luo et al [8] who also found that ChatGPT was not only used for self-reflection, finding a companion, or to improve mental health literacy, but also to actively manage mental health issues. This is a worrying trend considering general-purpose AI chatbots are not trained or certified to correctly and safely provide medical advice, potentially leading to misinformation and AI hallucinations [49].

### Reasons for Using AI Chatbots for Mental Health Support

The reasons for using AI chatbots for mental health seemed to parallel known barriers to mental health services, such as limited access, cost, and stigma, which is in line with previous research [6,33]. In both samples, most participants ranked constant availability and accessibility as the top reasons. The student sample also valued their anonymity, and the general sample reported feeling well-supported as an important reason for using them. Similar to prior work, people considered AI chatbots, mainly ChatGPT, to be helpful for their mental health [6,30,33]. Negative experiences were uncommon. Siddals et al [30] explored individuals’ experiences with generative AI chatbots for mental health by interviewing them. These interviews revealed that participants experienced high engagement and positive impacts, such as improved relationships and healing from trauma and loss. Notwithstanding their positive attitude, most students in our sample would not recommend AI chatbots for mental health. Considering their education, we hypothesize that applied psychology students value professional support over AI-based support. In contrast, the general population seemed more prone to recommending AI chatbot use for mental health. It is important to note, however, that perceived helpfulness does not mean that these general-purpose AI chatbots are actually effective in improving mental health conditions.

Prior research has consistently highlighted convenience, constant availability, and affordability as reasons for using AI chatbots for mental health [6,33]. It is therefore not surprising that generative AI models, such as general-purpose chatbot ChatGPT, are preferred by the public. This should, however, be a source of concern, as it remains unclear whether these general-purpose AI chatbots are safe and effective to use for mental health. Despite their benefits, LLMs can produce “hallucinations,” generating plausible but factually incorrect information caused by gaps in training data or flawed probabilistic reasoning. In addition, ethical concerns remain regarding the use of AI in mental health care, including issues of data privacy, algorithmic biases, and the risk of excessive reliance on technology without human oversight. Their lack of reliability, tendency to present inaccuracies with confidence, and potential for biases raise concerns, particularly in sensitive domains such as mental health [50,51]. Moreover, throughout the past years, popular media have reported multiple cases where prolonged AI chatbot interactions have had detrimental effects on people’s health, such as psychotic episodes [52] and even suicide [53]. Although these examples are rare, research has revealed that LLM-delivered therapy does not adhere to the same standards as conventional psychotherapy. Therapist empathy and genuineness are crucial contributors to the therapeutic relationship and resulting outcomes, and cannot be attributed to LLMs (even though responses may appear to show empathy) [54]. Despite their ability to generate coherent responses, LLMs may struggle with comprehending and addressing the complexities of mental health conditions compared to conventional approaches. For example, Moore et al [55] demonstrated that LLMs express stigma toward mental health conditions and respond inappropriately to common and realistic therapy situations. Similarly, another study, comparing responses to mental health scenarios by AI chatbots and therapists, revealed that general-purpose AI chatbots showed both elements of good therapy, such as validation and reassurance, but also overuse of directive advice without adequate prior inquiry and excessive use of overly generic interventions. The authors concluded that the latter made them unsuitable as therapy replacement, particularly in crisis situations [56]. Moreover, findings from 22 interviews with athletes showed that although the athletes reported improved mental health, reduced competition-related anxiety, and personalized coping strategies, they also experienced a lack of depth and repetitive responses, as well as insufficient cultural sensitivity [32]. Notwithstanding the constant evolution and improvements of the LLMs powering AI chatbots, research will first have to demonstrate the efficacy and safety of these chatbots when used for mental health.

### Working Alliance and Engagement

Although the concept of therapeutic or working alliance is considered a strong factor contributing to the success of in-person psychotherapy and guided mobile health interventions (ie, with human support) [25], evidence on (digital) working alliance with unguided digital mental health interventions (ie, without human support), such as AI chatbots, is limited. Two studies have shown that digital working alliance may promote engagement in unguided use of a smartphone-based meditation app, operationalized as frequency of use (ie, regular: daily or weekly; nonregular: monthly, several times per year, or never) [35] or automized app use (ie, days with in-app activity completion) [57]. To the best of our knowledge, our study revealed, for the first time, an association between digital working alliance and AI chatbot engagement, operationalized in the form of frequency (but not duration). We found that respondents in the general sample who reported a higher working alliance also used the AI chatbot more frequently. Note, however, that no causation is inferred. This association was not found for the duration of talking sessions with an AI chatbot. Xu et al [58] explored the concept of the digital therapeutic (or working) alliance with mental health chatbots via a 4-week diary study of individuals interacting with Wysa and Woebot. Results revealed that the participants were able to form a connection, called a bond or light bond. The researchers also identified 6 themes involved in the formation of these bonds (or lack thereof), reflecting both individual conversational preferences (ie, leading or being led in a conversation) and perceived effectiveness of the chatbot’s output [58]. More research into the concept of digital working alliance and its relation to digital mental health engagement is, however, needed.

### Implications for Practice

The findings from the present study show that general-purpose AI chatbots, specifically ChatGPT, are being used for obtaining social and emotional support, oftentimes by individuals already familiar with professional mental health support. It is, therefore, important for (aspiring) practitioners to educate themselves on which AI chatbots are available and how they function so that they can support, guide, advise, or protect their clients, if needed. Considering the finding that individuals often do not inform their therapist or counsellor about AI chatbot use, we also advise practitioners to actively ask their clients about AI chatbot use to better understand their (unmet) needs and functioning.

Aside from the practitioners, it is important to educate the general public about safe AI chatbot use on the one hand but also to inform them about the availability of evidence-based digital mental health tools on the other hand.

This fast-changing field also provides a lot of opportunities for researchers. The knowledge base is rapidly growing, yet comparisons between findings are difficult, as validated instruments or methods to assess LLMs and their effects on users are missing. In addition, it will be important to assess potential long-term effects of using LLMs for mental health support as well as the addition of AI in existing treatment plans.

Developers of AI chatbots should also be aware that individuals are already using such systems for mental health support and should put the necessary guardrails in place to guide users to more appropriate services when needed. Moreover, several frameworks already exist to aid developers to assess existing AI tools or support the creation of new AI tools for mental health support, for example, READI [59], CAPE [60], or a broader quality assessment framework for digital tools [61].

Policymakers are also encouraged to rethink health care organization. Current drawbacks of formal health care services include limited accessibility and high costs, which appears to push people toward using available AI systems as self-help tools. This is concerning since these most commonly available tools were not built with the purpose of handling and securing such potentially sensitive content. Rethinking how people access informal and formal mental health support and making sure systems and services align with public needs will be important work for inclusive and future-proof mental health systems [16].

### Limitations

Across both study samples, the adopted sampling strategy and study design might have led to different types of bias. Because participants were recruited using convenience sampling, the sample may not be representative of the target population, introducing selection bias and limiting the generalizability of the findings. Convenience sampling of individuals already starting to use AI chatbots for mental health support might have led to participation of individuals with strong opinions on the matter, potentially biasing the findings. As the data were self-reported and no fixed recall period was appointed, recall bias cannot be excluded.

The majority of student participants indicated that they seldom engaged in mental health-focused conversations with AI chatbots, which may have resulted in their comparatively superficial responses outweighing those of participants who regularly use AI for such interactions. In addition, convenience sampling in the general population may have introduced some bias in our sample, considering our recruitment strategy might have been more prone to attract female participants (eg, via magazines).

No information on ethnicity or socioeconomic status was collected, limiting deeper insights into the respondents. In addition, the lack of in-depth questions on potential negative experiences limits further insight into the specific potential dangers, such as misinformation. Moreover, no clinical outcome measures were collected, which warrants caution in interpreting the respondents’ reported helpfulness of AI chatbots. Despite the acceptable reliability of the translated D-WAI, findings should be used with caution as no formal psychometric validation was undertaken for this translated version. In addition, participants were not instructed to have a particular AI chatbot in mind. Therefore, it is unclear to which AI chatbot the working alliance refers to if they have been using multiple chatbots for support. Following studies should specify this clearly when assessing working alliance. Future studies would benefit from larger and more diverse samples to make between-group comparisons possible and to improve generalizability. Finally, there is no universal definition in use for mental health support in the context of AI mental health research [44]. This makes comparing findings between studies difficult.

Future work can build on the current study and would benefit from additional studies in large, representative samples adopting longitudinal designs with clinical outcome measures to assess the potential effects of continued use of AI chatbots, either general-purpose or regulated for mental health support. Finally, further assessment of digital working alliance with general-purpose and regulated mental health AI chatbots is needed to better understand and envisage how AI chatbots could be used (or not) for mental health support.

### Conclusion

This study confirms that general-purpose AI chatbots that are not designed or regulated for mental health support, specifically ChatGPT, are being used for obtaining social and emotional support, oftentimes by individuals already familiar with professional mental health support. Respondents generally perceived the conversations as helpful, although no clinical outcomes were assessed, and there were few reported negative experiences. AI chatbots were mostly appealing because of their availability and accessibility. Finally, the preliminary association between digital working alliance and engagement factors such as frequency and duration highlighted that relational factors might play a role in sustained AI chatbot use but warrant further investigation.

## Appendix

Multimedia Appendix 1 Adopted survey in Dutch and English.

## Abbreviations

D-WAI: Digital Working Alliance Inventory
LLM: large language model

## Data Availability

The data sets generated and/or analyzed during this study are available from the corresponding author on reasonable request.

## References

[1] Stryker C What are large language models (LLMs)?. IBM.

[2] Chatterji Aaron, Cunningham Thomas, Deming David J., Hitzig Zoe, Ong Christopher, Shan Carl Yan, Wadman Kevin (2025). How people use chatGPT. National Bureau of Economic Research Working Paper 34255.

[3] How people ask claude for personal guidance. Anthropic.

[4] Scherr S, Cao B, Jiang LC, Kobayashi T (2025). Explaining the use of AI chatbots as context alignment: motivations behind the use of AI chatbots across contexts and culture. Comput Hum Behav.

[5] Rousmaniere T, Zhang Y, Li X, Shah S (2026). Large language models as mental health resources: patterns of use in the United States. Pract Innov.

[6] Stade Elizabeth C, Tait Zoe M, Campione Samuel T, Wiltsey Stirman Shannon, Eichstaedt Johannes C (2026). Real-world use of large language models for mental health in 2024. NPJ Digit Med.

[7] Americal Psychological Association (2025). APA Health Advisory on the Use of Generative AI Chatbots and Wellness Applications for Mental Healt.

[8] Luo X, Ghosh S, Tilley JL, Besada P, Wang J, Xiang Y (2025). "Shaping ChatGPT into my Digital Therapist": a thematic analysis of social media discourse on using generative artificial intelligence for mental health. Digit Health.

[9] Li Z, Zhu Z, Gui X, Luo Y (2025). “This is human intelligence debugging artificial intelligence”: examining how people prompt GPT in seeking mental health support. Int J Hum Comput Stud.

[10] Sorin V, Brin D, Barash Y, Konen E, Charney A, Nadkarni G, Klang E (2024). Large language models and empathy: systematic review. J Med Internet Res.

[11] Monteith S, Glenn T, Geddes JR, Whybrow PC, Achtyes E, Bauer M (2026). Anthropomorphic technology in everyday life: focus on chatbots and impacts on mental health. Eur Arch Psychiatry Clin Neurosci.

[12] Cheng M, Lee C, Khadpe P, Yu S, Han D, Jurafsky D (2026). Sycophantic AI decreases prosocial intentions and promotes dependence. Science.

[13] Ueda M, Birnbaum ML, Liu Y, Yu Q, Tian X, Mirer A, Ramanathan S, Sinyor M (2026). Help-seeking in the age of AI: cross-sectional survey of the use and perceptions of AI-based mental health support among US adults. JMIR Ment Health.

[14] Andary S, Bassani J, Burrell G, Cole E, Evans R, Redman E, Kumar S (2023). Barriers and enablers to access and utilization of mental health care services across Southeast Asia: a preliminary scoping review. Asia Pac Psychiatry.

[15] Moroz N, Moroz I, D'Angelo Monika Slovinec (2020). Mental health services in Canada: barriers and cost-effective solutions to increase access. Healthc Manage Forum.

[16] De Witte NAJ, Best P, Torous J, Mulvenna M, Van Assche E, Mathiasen K, Kleiboer A, Carlbring P, Van Daele T (2026). Comprehensive model for mental health access and service use (CoMMA): a process model for technology-enhanced mental healthcare. Internet Interv.

[17] Gaffney H, Mansell W, Tai S (2019). Conversational agents in the treatment of mental health problems: mixed-method systematic review. JMIR Ment Health.

[18] Vaidyam AN, Linggonegoro D, Torous J (2021). Changes to the psychiatric chatbot landscape: A systematic review of conversational agents in serious mental Illness: Changements du paysage psychiatrique des chatbots: une revue systématique des agents conversationnels dans la maladie mentale sérieuse. Can J Psychiatry.

[19] Hua Y, Siddals S, Ma Z, Galatzer-Levy I, Xia W, Hau C, Na H, Flathers M, Linardon J, Ayubcha C, Torous J (2025). Charting the evolution of artificial intelligence mental health chatbots from rule-based systems to large language models: a systematic review. World Psychiatry.

[20] Feng X, Tian L, Ho GWK, Yorke J, Hui V (2025). The effectiveness of AI chatbots in alleviating mental distress and promoting health behaviors among adolescents and young adults: systematic review and meta-analysis. J Med Internet Res.

[21] Heinz MV, Mackin DM, Trudeau BM, Bhattacharya S, Wang Y, Banta HA, Jewett AD, Salzhauer AJ, Griffin TZ, Jacobson NC (2025). Randomized trial of a generative AI chatbot for mental health treatment. NEJM AI.

[22] Zhong W, Luo J, Zhang H (2024). The therapeutic effectiveness of artificial intelligence-based chatbots in alleviation of depressive and anxiety symptoms in short-course treatments: a systematic review and meta-analysis. J Affect Disord.

[23] Farzan M, Ebrahimi H, Pourali M, Sabeti F (2025). Artificial intelligence-powered cognitive behavioral therapy chatbots, a systematic review. Iran J Psychiatry.

[24] Bodner R, Lim K, Schneider R, Torous J (2026). Efficacy and risks of artificial intelligence chatbots for anxiety and depression: a narrative review of recent clinical studies. Curr Opin Psychiatry.

[25] Flückiger C, Del Re AC, Wampold BE, Horvath AO (2018). The alliance in adult psychotherapy: a meta-analytic synthesis. Psychotherapy (Chic).

[26] Macrynikola N, Chang S, Torous J (2025). Is digital alliance associated with engagement & outcomes in guided digital interventions? An analysis of data from two studies. J Affect Disord.

[27] Rahsepar Meadi M, Sillekens T, Metselaar S, van Balkom A, Bernstein J, Batelaan N (2025). Exploring the ethical challenges of conversational AI in mental health care: scoping review. JMIR Ment Health.

[28] Rackoff GN, Zhang ZZ, Newman MG (2025). Chatbot-delivered mental health support: attitudes and utilization in a sample of U.S. college students. Digit Health.

[29] Alanezi F (2024). Assessing the effectiveness of ChatGPT in delivering mental health support: a qualitative study. J Multidiscip Healthc.

[30] Siddals S, Torous J, Coxon A (2024). "It happened to be the perfect thing": Experiences of generative AI chatbots for mental health. Npj Ment Health Res.

[31] Song I, Pendse S, Kumar N, De CM (2026). The typing cure: experiences with large language model chatbots for mental health support. arXiv.

[32] Maisarah S, Lee A, Azzahranisa N (2025). Investigating the user experience of AI chatbots in delivering mental health support to athletes. Health Nexus.

[33] Zaia Sam, Huthwaite Mark, Mathieson Fiona (2025). Perceived benefits and limitations of a generative AI chatbot for mental health support: an exploratory mixed-methods study. N Z Med Stud J.

[34] Henson P, Wisniewski H, Hollis C, Keshavan M, Torous J (2019). Digital mental health apps and the therapeutic alliance: initial review. BJPsych Open.

[35] Goldberg SB, Baldwin SA, Riordan KM, Torous J, Dahl CJ, Davidson RJ, Hirshberg MJ (2022). Alliance with an unguided smartphone app: validation of the digital working alliance inventory. Assessment.

[36] Wickham H, François R, Henry L, Müller K, Vaughan D (2023). A Grammar of Data Manipulation. dplyr.

[37] Wickham H (2025). Simple, Consistent Wrappers for Common String Operations. Stringr.

[38] Wickham H Elegant Graphics for Data Analysis. Ggplot2.

[39] Wilke CO (2025). Streamlined Plot Theme and Plot Annotations for “ggplot2. cowplot.

[40] Wickham H, Bryan J Read Excel Files. readxl.

[41] Wickham H, Vaughan D, Girlich M, Ushey K, Software P, PBC (2025). Tidy Messy Data. tidyr.

[42] Li L, Peng W, Rheu MM (2024). Factors predicting intentions of adoption and continued use of artificial intelligence chatbots for mental health: examining the role of UTAUT model, stigma, privacy concerns, and artificial intelligence hesitancy. Telemed J E Health.

[43] (2025). MIND report 'Concerning or promising? Generative AI for mental health issues'. MIND.

[44] Bodner R, Lim K, Siddals S, Goldberg S, Torous J (2026). Barriers to understanding how many people use AI for mental health support: an estimate and narrative review. npj Digit Public Health.

[45] Essential snapchat statistics you need to know in 2026. The Social Shepherd.

[46] Fitzpatrick KK, Darcy A, Vierhile M (2017). Delivering cognitive behavior therapy to young adults with symptoms of depression and anxiety using a fully automated conversational agent (Woebot): a randomized controlled trial. JMIR Ment Health.

[47] Fulmer R, Joerin A, Gentile B, Lakerink L, Rauws M (2018). Using psychological artificial intelligence (Tess) to relieve symptoms of depression and anxiety: randomized controlled trial. JMIR Ment Health.

[48] MacNeill AL, Doucet S, Luke A (2024). Effectiveness of a mental health chatbot for people with chronic diseases: randomized controlled trial. JMIR Form Res.

[49] Aydin S, Karabacak M, Vlachos V, Margetis K (2024). Large language models in patient education: a scoping review of applications in medicine. Front Med (Lausanne).

[50] Wang X, Zhou Y, Zhou G (2025). The application and ethical implication of generative AI in mental health: systematic review. JMIR Ment Health.

[51] Wang L, Bhanushali T, Huang Z, Yang J, Badami S, Hightow-Weidman L (2025). Evaluating generative AI in mental health: systematic review of capabilities and limitations. JMIR Ment Health.

[52] Keshavan M, Torous J, Yassin W (2026). Do generative AI chatbots increase psychosis risk?. World Psychiatry.

[53] Stokel-Walker C (2025). AI driven psychosis and suicide are on the rise, but what happens if we turn the chatbots off?. BMJ.

[54] Nienhuis JB, Owen J, Valentine JC, Winkeljohn Black S, Halford TC, Parazak SE, Budge S, Hilsenroth M (2018). Therapeutic alliance, empathy, and genuineness in individual adult psychotherapy: a meta-analytic review. Psychother Res.

[55] Moore J, Grabb D, Agnew W (2025). Expressing stigma and inappropriate responses prevents LLMs from safely replacing mental health providers.

[56] Scholich T, Barr M, Wiltsey Stirman S, Raj S (2025). A comparison of responses from human therapists and large language model-based chatbots to assess therapeutic communication: mixed methods study. JMIR Ment Health.

[57] Goldberg SB, Jiwani Z, Bolt DM, Riordan KM, Davidson RJ, Hirshberg MJ (2024). Evidence for bidirectional, cross-lagged associations between alliance and psychological distress in an unguided mobile-health intervention. Clin Psychol Sci.

[58] Xu Z, Lee Y, Stasiak K, Warren J, Lottridge D (2025). The digital therapeutic alliance with mental health chatbots: diary study and thematic analysis. JMIR Ment Health.

[59] Stade EC, Eichstaedt JC, Kim JP, Stirman SW (2025). Readiness evaluation for AI-mental health deployment and implementation (READI): a review and proposed framework. Technol Mind Behav.

[60] Sobowale K, Humphrey DK (2025). Evaluating the quality of psychotherapy conversational agents: framework development and cross-sectional study. JMIR Form Res.

[61] Buelens F, Seymoens T, Verplancke J, Van Daele T (2026). Development of a contextualized, research-based flemish assessment framework for digital care, assistance, and support: Delphi study. JMIR Form Res.

