# Supplementary material for: Exploring Real-World Use of AI Chatbots for Mental Health Support: Cross-Sectional Survey Study
Source: JMIR Ment Health. 2026 Sep 1;13:e104316. doi: 10.2196/104316 (PMC13579130; doi:10.2196/104316)
Supplement: Supplementary file 1 [file mental_v13i1e104316_app1.pdf]

*

THOMAS MORE

Chatbots voor mentaal welzijn

Doel van het onderzoek

Steeds meer mensen lijken AI-chatbots niet alleen als bron van informatie of voor hun werk of opleiding te gebruiken, maar ook als steun voor hun mentaal welzijn. Soms als luisterend oor, soms als gezelschap, soms als aanvulling op professionele hulp. Met deze bevraging willen we beter begrijpen hoe mensen chatbots hier effectief voor gebruiken, welke ervaringen ze hebben, en wat hun verwachtingen zijn. We zijn daarbij enkel geïnteresseerd in het speciŁeke gebruik ervan voor mentale steun zoals bijvoorbeeld praten over persoonlijke zorgen of problemen, het bespreken van je sociaal leven, gesprekken over intimiteit of seksualiteit, of als gezelschap of om je minder alleen te voelen.

Praktisch

De online bevraging duurt ongeveer 5 minuten en staat open voor iedereen van zestien jaar of ouder. Thomas More-hogeschool respecteert alle wettelijke bepalingen inzake de bescherming van de persoonlijke gegevens van de ondervraagde personen (de Algemene Verordening Gegevensbescherming van 24 mei 2016 en andere van toepassing zijnde wetgeving m.b.t. gegevensbescherming). Thomas More verwerkt jouw persoonsgegevens conform de privacyverklaring onderzoek en dienstverlening van Thomas More-hogeschool.

Contactgegevens onderzoekers
Tom Van Daele, Sylvie Bernaerts, Fien Buelens, Nele De Witte, Eva Van Assche, & Toon Colman via experiencehub@thomasmore.be

Download de volledige informatiebrief voor meer achtergrond.

- ◯ Ik heb bovenstaande informatie gelezen en begrepen. Ik stem toe om deel te nemen.
- ◯ Ik wens niet deel te nemen.

*** Ben je zestien jaar of ouder en heb je al eens een chatbot gebruikt voor je mentaal welzijn?**

Daarmee bedoelen we onder meer praten over persoonlijke zorgen of problemen, het bespreken van je sociaal leven, gesprekken over intimiteit of seksualiteit, of als gezelschap of je minder alleen te voelen.

- ◯ Ja
- ◯ Nee

***Hoe gebruik je chatbots voor je mentaal welzijn?**
meerdere antwoorden mogelijk

- ☐ Om te praten over persoonlijke zorgen of problemen
- ☐ Om situaties in mijn sociale leven te bespreken
- ☐ Om te praten over intimiteit of seksualiteit
- ☐ Als gezelschap of om me minder alleen te voelen
- ☐ Anders, namelijk:

***Welke chatbot(s) heb je hiervoor gebruikt?**

meerdere antwoorden mogelijk

- ☐ ChatGPT
- ☐ Gemini
- ☐ DeepSeek
- ☐ Claude
- ☐ Perplexity
- ☐ Copilot
- ☐ Grok
- ☐ My AI - Snapchat
- ☐ Replika
- ☐ Character AI
- ☐ Andere, namelijk:

▫ Heb je zelf ooit al betaald voor het gebruik van een chatbot voor je mentaal welzijn?

- ○ Nee, ik gebruik enkel gratis versies
- ○ Nee, maar via mijn werk of opleiding heb ik wel toegang tot een betalend abonnement dat ik ook daarvoor gebruik.
- ○ Ja, ik heb hier persoonlijk al voor betaald

***Op welk toestel praat je meestal met chatbots over je mentaal welzijn?**
meerdere antwoorden mogelijk

- ☐ Smartphone
- ☐ PC

☐ Tablet

*** Op welke manier praat je met chatbots over je mentaal welzijn?**

◯ Alleen via tekst

◯ Alleen via stem

◯ Een combinatie van tekst en stem

*** Hoe vaak praat je met chatbots over je mentaal welzijn?**

◯ Dagelijks

◯ Enkele keren per week

◯ Enkele keren per maand

◯ Zelden

*** Hoe lang duren je gesprekken rond je mentaal welzijn met een chatbot gemiddeld?**

◯ Minder dan 5 minuten

◯ 5 - 15 minuten

◯ 15 - 30 minuten

◯ Meer dan 30 minuten

*** Sinds wanneer voer je ongeveer gesprekken rond je mentaal welzijn met een chatbot?**

◯ Sinds minder dan een maand

◯ Sinds 3 maanden

- ◯ Sinds een half jaar
- ◯ Sinds een jaar
- ◯ Langer dan een jaar

***Waarom gebruik je chatbots voor mentaal welzijn?**
meerdere antwoorden mogelijk

- ☐ Chatbots zijn altijd en overal voor mij beschikbaar.
- ☐ Chatbots bieden me goede ondersteuning.
- ☐ Chatbots bieden me anonimiteit.
- ☐ Chatbots zijn laagdrempelig: ik vind het gemakkelijker om met een chatbot te praten dan met een echt persoon
- ☐ Chatbots worden me aanbevolen door mensen uit mijn omgeving.
- ☐ Chatbots zijn gratis of goedkoper dan bestaande hulpverlening.
- ☐ Ik vind praten met chatbots leuk.
- ☐ Ik gebruik chatbots voor alles, ook om over mijn mentaal welzijn te praten.
- ☐ Een andere reden, namelijk:

***In welke mate vind je gesprekken rond je mentaal welzijn met chatbots helpend?**

- ◯ Helemaal niet helpend
- ◯ Eerder niet helpend
- ◯ Neutraal
- ◯ Eerder helpend
- ◯ Heel helpend

*** Zou je het gebruik van chatbots rond je mentaal welzijn aanraden aan anderen?**

- ◯ Zeker niet
- ◯ Waarschijnlijk niet
- ◯ Misschien / weet ik niet
- ◯ Waarschijnlijk wel
- ◯ Zeker wel

*** Heb je ooit al negatieve ervaringen gehad met een chatbot rond je mentaal welzijn?**

- ◯ Ja
- ◯ Nee

Kan je hier kort beschrijven welke negatieve ervaring je hebt gehad?

* De volgende vragen hebben betrekking op hoe je je gesprekken met een chatbot ziet, wanneer je ermee spreekt over je mentaal welzijn.

	Helemaal niet akkoord	Niet akkoord	Enigszins niet akkoord	Neutraal	Enigszins akkoord	Akkoord	Helemaal akkoord
Ik vertrouw erop dat de chatbot me naar mijn persoonlijke doelen zal begeleiden.	◯	◯	◯	◯	◯	◯	◯
Ik geloof dat de chatbot me zal helpen om mijn probleem aan te pakken.	◯	◯	◯	◯	◯	◯	◯

De chatbot moedigt me aan om taken te volbrengen en vooruitgang te boeken.	◯	◯	◯	◯	◯	◯	◯
Ik ben het ermee eens dat aanbevelingen van de chatbot belangrijk zijn voor mijn doelen.	◯	◯	◯	◯	◯	◯	◯
De chatbot is gemakkelijk om te gebruiken.	◯	◯	◯	◯	◯	◯	◯
De chatbot ondersteunt me om uitdagingen te overwinnen.	◯	◯	◯	◯	◯	◯	◯

*** Ben je in behandeling bij een psycholoog, psychiater of andere hulpverlener?**

◯ Ik ben momenteel in behandeling.

◯ Ik ben in behandeling geweest.

◯ Ik heb geen ervaring met behandeling.

*** Is je hulpverlener ervan op de hoogte dat je ook een chatbot gebruikt om te praten over je mentaal welzijn?**

◯ Ja

◯ Nee

*** Was je hulpverlener ervan op de hoogte dat je ook een chatbot gebruikte om te praten over je mentaal welzijn?**

◯ Ja

◯ Nee

- Ik ben ...... jaar oud.

*** Hoe zie je jezelf? Ik zie mezelf als een**

- ◯ Vrouw
- ◯ Man
- ◯ Ik wil liever niet antwoorden
- ◯ Anders, namelijk

*** Wat is de hoogste graad die je op dit moment hebt behaald?**

- ◯ Lager onderwijs
- ◯ Secundair onderwijs
- ◯ Academische of Professionele bachelor
- ◯ Master
- ◯ Doctoraat

Wil je graag nog iets delen over dit onderwerp of deze bevraging? Dan kan dit hier.

Zou je bereid zijn om in een kort online interview meer te vertellen over je ervaring?

Indien je interesse hebt om je mening te delen, word je vrijblijvend

gecontacteerd met meer informatie.
Resultaten worden ook hier steeds anoniem gecommuniceerd.

- ◯ Ja, je mag me contacteren.
- ◯ Nee, liever niet.

*

Mental Wellness Chatbots

Purpose of the Study

More and more people seem to be using AI chatbots not only to find information or for work or school, but also to support their mental well-being. The AI chatbot c,an lend an ear, provide companionship, and can sometimes act as a supplement to professional help. With this survey, we want to better understand how people actually use chatbots for mental health purposes, what their experiences are, and what they expect. We're only interested in their specific use for mental support, for example, talking about personal worries or problems, discussing your social life, conversations about intimacy or sexuality, or using a chatbot for companionship or to feel less alone.

Practical Information

The online survey takes about 5 minutes and is open to anyone aged 16 or older. Thomas More University of Applied Sciences complies with all legal requirements for protecting the personal data of survey participants (the General Data Protection Regulation of May 24, 2016, and other applicable data-protection laws). Thomas More processes your personal data in line with the Research and Services Privacy Statement of Thomas More University of Applied Sciences.

Research team contact info
Tom Van Daele, Sylvie Bernaerts, Fien Buelens, Nele De Witte, Eva Van Assche, & Toon Colman at experiencehub@thomasmore.be

Download the full information letter for more background.

- ◯ I have read and understood the information above. I agree to participate.
- ◯ I do not wish to participate.

*** Are you 16 or older, and have you ever used a chatbot to support your mental well-being?**

By that, we mean things such as talking through personal worries or problems, discussing situations in your social life, conversations about intimacy or sexuality, or using it for companionship so you feel less alone.

- ◯ Yes
- ◯ No

*** How do you use chatbots for your mental well-being?** select all that apply

- ☐ To talk about personal worries or problems
- ☐ To discuss situations in my social life
- ☐ To talk about intimacy or sexuality
- ☐ For companionship or to feel less alone
- ☐ Other, please specify:

*** Which chatbot(s) did you use for this?**

multiple answers allowed

- ☐ ChatGPT
- ☐ Gemini
- ☐ DeepSeek
- ☐ Claude
- ☐ Perplexity
- ☐ Copilot
- ☐ Grok
- ☐ My AI - Snapchat
- ☐ Replika
- ☐ Character AI
- ☐ Other, namely:

▫ Have you ever paid to use a chatbot for your mental wellness?

- ◯ No, I only use free versions
- ◯ No, but through my job or school I do have access to a paid subscription that I also use for that.
- ◯ Yes, I've paid for this myself

*__Which device do you usually use to chat with chatbots about your mental well-being?__ Multiple answers allowed

- ☐ Smartphone
- ☐ PC

- [ ] Tablet

*How do you talk with chatbots about your mental well-being?

- ○ Text only
- ○ Voice only
- ○ A mix of text and voice

***How often do you talk with chatbots about your mental well-being?**

- ○ Daily
- ○ A few times a week
- ○ A few times a month
- ○ Rarely

*On average, how long are your mental well-being conversations with a chatbot?

- ○ Less than 5 minutes
- ○ 5–15 minutes
- ○ 15–30 minutes
- ○ More than 30 minutes

*About how long have you been having mental well-being conversations with a chatbot?

- ○ Less than a month
- ○ About 3 months

- For about six months
- For a year
- Longer than a year

***Why do you use AI chatbots for mental well-being?**
Select all that apply

- Chatbots are available to me anytime, anywhere.
- Chatbots give me solid support.
- Chatbots let me stay anonymous.
- Chatbots feel low-pressure: it’s easier for me to talk to a chatbot than to a real person.
- People around me recommend chatbots to me.
- Chatbots are free to use, or cheaper than traditional support services.
- I enjoy talking with chatbots.
- I use chatbots for pretty much everything, including talking about my mental well-being.
- Another reason, namely:

***How helpful do you find conversations with chatbots about your mental wellness?**

- Not helpful at all
- Somewhat unhelpful
- Neutral
- Somewhat helpful
- Very helpful

* Would you recommend using chatbots for mental well-being to others?

- ◯ Definitely not
- ◯ Probably not
- ◯ Maybe / I don’t know
- ◯ Probably
- ◯ Definitely

* Have you ever had a negative experience with a chatbot about your mental well-being?

- ◯ Yes
- ◯ No

Could you briefly describe the negative experience you had?

* The following questions are about how you view your conversations with a chatbot when you talk about your mental well-being.

	Strongly disagree	Dis agree	Somewhat disagree	Neutral	Somewhat agree	Agree	Strongly agree
I trust the chatbot to guide me toward my personal goals.	◯	◯	◯	◯	◯	◯	◯
I believe the chatbot will help me deal with my problem.	◯	◯	◯	◯	◯	◯	◯

The chatbot encourages me to complete tasks and keep making progress.	◯	◯	◯	◯	◯	◯	◯
I agree that the chatbot's suggestions are important for reaching my goals.	◯	◯	◯	◯	◯	◯	◯
The chatbot is easy to use.	◯	◯	◯	◯	◯	◯	◯
The chatbot helps me work through challenges.	◯	◯	◯	◯	◯	◯	◯

* Are you currently seeing a psychologist, psychiatrist, or another mental health professional?

◯ I'm currently receiving treatment.

◯ I've received treatment in the past.

◯ I don't have any experience with treatment.

* Does your provider know that you also use a chatbot to talk about your mental well-being?

◯ Yes

◯ No

* Did your provider know that you were also using a chatbot to talk about your mental well-being?

◯ Yes

◯ No

- I am ...... years old.

*** How do you identify? I identify as a**

- ◯ Woman
- ◯ Man
- ◯ I’d rather not answer
- ◯ Other, namely

*** What is the highest degree you have completed so far?**

- ◯ Elementary school
- ◯ High school
- ◯ Academic or Professional Bachelor’s degree
- ◯ Master’s degree
- ◯ Doctorate (PhD)

Would you like to share anything else about this topic or this survey? You can do so here.

Would you be willing to share more about your experience in a brief online interview?

If you’re interested in sharing your thoughts, you’re welcome to do so with no obligation

contacted with more information.
Results are always shared anonymously here as well.

- ◯ Yes, you can contact me.
- ◯ No, I’d rather not.

QuestionPro
